# A Case of Late Cardiac Tamponade After a Complex Percutaneous Coronary Intervention

**DOI:** 10.7759/cureus.43700

**Published:** 2023-08-18

**Authors:** Fahad B Tahir, Sualeha Zulfiqar, Muhammad Umair Rana, Fawad Haroon

**Affiliations:** 1 Hospital Medicine, TidalHealth Peninsula Regional, Salisbury, USA; 2 Emergency, Mayo Hospital, Lahore, PAK; 3 Cardiology, Carle Foundation Hospital, Urbana, USA

**Keywords:** coronary artery perforation, complex pci, rotational atherectomy, pericardial effusion, tamponade

## Abstract

Rotational atherectomy (RA) is an endovascular procedure to ablate calcified plaque and is an integral tool for complex percutaneous coronary intervention (PCI). Rotational atherectomy increases the risk of periprocedural complications. One of the major complications of RA is coronary perforation, which has a reported incidence of up to 2%. It is usually identified and managed within the procedure. Rarely, there are delayed and unanticipated complications that can be missed, causing significant morbidity and mortality. We present a rare case of a patient with a late presentation of cardiac tamponade days after a complex PCI with RA.

## Introduction

Rotational atherectomy (RA) is an athero-ablative technique to break up calcified plaque that is clogging a coronary artery. It operates on the mechanism of ‘differential cutting’ and ablates hard and calcified plaque using a diamond-encrusted elliptical burr [1,2]. The reported incidence of coronary perforation rates [3] in PCI with RA is higher (2%) than in percutaneous coronary intervention (PCI) without RA (0.4%). Patients may need emergent pericardiocentesis in case cardiac tamponade develops as a complication. Late complications are unusual and have been infrequently described in the literature. Here, we present a rare instance of a patient who presented with cardiac tamponade days after RA and PCI.

## Case presentation

**Figure 1:**
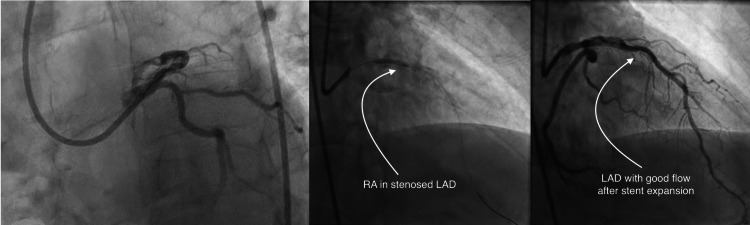
Cardiac catheterization demonstrating the complex PCI with rotational atherectomy procedure. PCI: percutaneous coronary intervention, LAD: left anterior descending artery, RA: rotational atherectomy.

**Figure 2:**
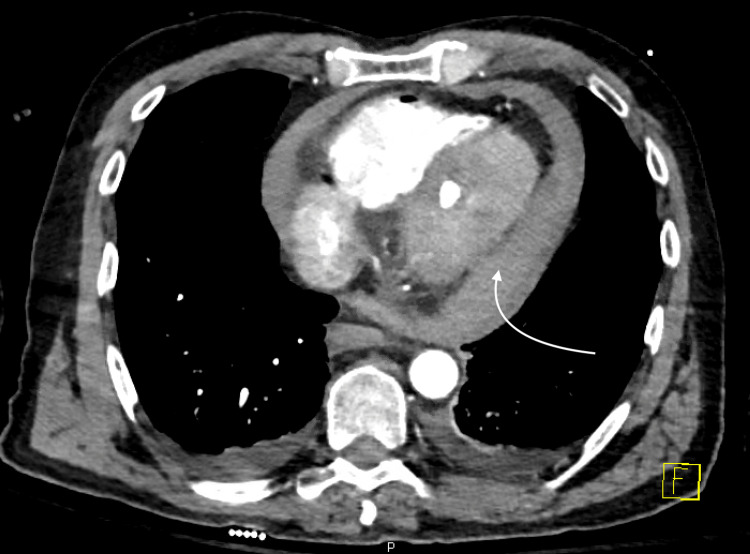
Computed tomography angiography (CTA) chest demonstrating a moderate-size pericardial effusion highlighted by the arrow.

**Figure 3:**
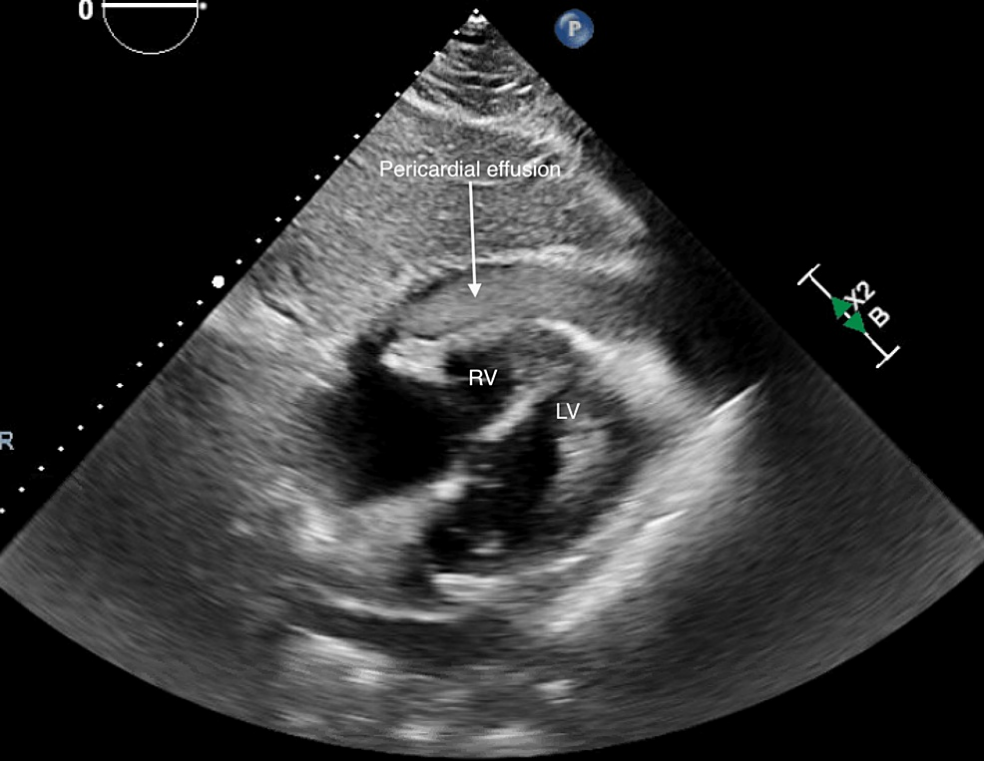
Transthoracic apical echocardiogram demonstrating a hyperdense pericardial effusion, as indicated by the arrow in the figure. LV: left ventricle, RV: right ventricle.

A 62-year-old man was initially admitted with chest pain and emergently taken to the cardiac catheterization lab, where he was noted to have acute thrombotic occlusion of the right coronary artery (RCA). Successful percutaneous transluminal coronary angioplasty (PTCA) was undertaken; however, the vessel caliber was too small to accommodate a stent. Additionally, the patient was noted to have chronic calcified disease in the proximal left anterior descending artery (LAD). The patient underwent a second cardiac catheterization two days later, having a rotational atherectomy and PCI to the LAD with three drug-eluting stents (Figure 1). The patient was hemodynamically stable post-procedure and discharged home the next day with dual antiplatelet therapy (DAPT). An echocardiogram prior to discharge noted a small pericardial effusion with no tamponade physiology. Two days after the discharge, the patient presented again with chest pain and hypotension. Computed tomography angiography (CTA) of the chest (Figure 2) noted a hyperdense pericardial effusion, and the STAT echocardiogram (Figure 3) was concerning for hemopericardium with tamponade. Both interventional cardiology and cardiothoracic surgery were urgently consulted, and after discussion among specialties, the decision was taken to manage with a pericardial window instead of pericardiocentesis, considering the suspicion for clotted blood in the pericardial space. He was taken to the cardio-thoracic surgery theater and underwent a subxiphoid pericardial window, evacuating approximately 500 cc of dark red blood and clots. Upon evacuating the pericardium, the patient's hemodynamics improved significantly. DAPT was held on the day of the procedure. The next day, he was restarted on DAPT and discharged home with a plan for close follow-up. He remained stable and was evaluated at the outpatient follow-up in one week.

## Discussion

The percutaneous coronary intervention of calcified coronary plaques remains an interventional challenge for cardiologists. RA is a technique used to prepare the calcified plaques before stent expansion [4]. It is an operator- and team-dependent procedure, and its use is limited to hard calcified plaques only. The ROTAXUS study on a population needing routine PCI found no significant difference in major adverse cardiac events and stent restenosis at nine months between the rotablation plus PCI vs PCI alone groups. Complications of RA are rare, usually peri-procedural and include slow-flow/no-reflow, burr entrapment, and coronary dissection and perforation [5]. If a coronary perforation is identified, RA is usually stopped and the priority is to maintain the wire inside the true lumen and complete the PCI with balloon angioplasty and stenting expeditiously. Management strategies for coronary perforation include balloon tamponade, coil embolization, and covered stents. RA operators should be proficient in emergent pericardiocentesis, which can be lifesaving in case cardiac tamponade develops. Cardiothoracic surgery consultation for a pericardial window may be required. The only major delayed complication for RA is stent restenosis and in-stent late lumen loss. Based on our case, a subacute complication, i.e., pericardial effusion with potential cardiac tamponade, should be recognized as well.

## Conclusions

Rotational atherectomy is a procedure to break inelastic plaque used in cases where a stent cannot be reliably delivered to the target. Complications of rotational atherectomy, although rare, can carry significant morbidity and mortality. Late cardiac tamponade is an unrecognized complication of RA/complex PCI. We propose that after RA, mild or moderate effusion on echocardiogram should be followed for hemodynamic significance, and delayed hemopericardium should be in the differential in cases of recurrent chest discomfort or shortness of breath in patients with complex PCI. Management would include holding or reversing anticoagulation and emergent pericardiocentesis in case the patient develops a tamponade. 
